# Labour market exits in a former out-of-home care population: A birth cohort-based sequence analysis

**DOI:** 10.1016/j.ssmph.2025.101885

**Published:** 2025-11-15

**Authors:** Lisa Bornscheuer, Josephine Jackisch, Karl Gauffin, Ylva B. Almquist

**Affiliations:** a Department of Public Health Sciences, Stockholm University, Albanovägen 12, Stockholm, SE 106 91, Sweden; b Max Planck Institute for Demographic Research, Konrad-Zuse Str.1, Rostock, 18057, Germany

**Keywords:** Childhood adversity, Out-of-home care, Retirement, Labour market exit, Sequence analysis

## Abstract

Ways of exiting the labour market both reflect previous health and socioeconomic disadvantage, and shape opportunities for healthy ageing. We apply sequence analysis on data from a 1953 Stockholm birth cohort to describe typical labour market exit routes between ages 55 and 68, both in the full sample and among the subgroup of individuals with childhood experience of out-of-home care for family reasons — a population with a high prevalence of childhood adversity. Information on income is used to further characterize these routes. Based on multinomial logistic regression analysis, we examine educational attainment and gender as predictors of exit routes, and as effect modifiers in the association between out-of-home care and exit routes. The normative transition from employment to pension was the most common type of exit in both samples. Individuals clustered into two non-normative routes in the full sample (health-related benefits; early mortality) and four non-normative routes in the care-experienced sample (health-related benefits with income from work; health-related benefits without income from work; unemployment; early mortality), largely reflecting a higher degree of financial disadvantage. We furthermore show that out-of-home care is associated with higher odds of following non-normative exit routes and that higher educational attainment might have the potential to mitigate this association. No consistent differences between men and women emerged in the analyses. Altogether, this study is the first to take a person-centred and prospective approach to describe the heterogeneity in early labour market exits in a high-risk population. Future research should further explore resilience factors in this context.

## Highlights

• Non-normative labour market exits reflect health and socioeconomic disadvantage.
• Individuals with experience of out-of-home care placement have a higher risk of non-normative exit routes.
• Still, two-thirds follow normative exit routes, which can be seen as indicative of underlying resilience processes.
• Educational attainment contributes to resilience.

## 1 Introduction

Children placed in out-of-home care (OHC) are likely to have experienced substantial adversity early on (Jackisch et al., 2019). Although OHC is intended to provide these children with improved life chances, research has consistently found them to be at greater risk of long-term health and socioeconomic disadvantages. Importantly, these disadvantages tend to be multidimensional, mutually reinforcing, and cumulative (Almquist, 2016; Brännström, Vinnerljung, et al., 2017; Hoffmann et al., 2018). Weak labour market attachment is one such outcome, and is more common among former OHC populations (Kääriälä et al., 2019). Long-term or repeated periods of unemployment and sick leave can simultaneously signal health and socioeconomic disadvantage, and play an important part in shaping labour market exit routes. Furthermore, early labour market exits are a socially stratified phenomenon, commonly patterned by gender and education (Carlsson et al., 2023; Madero-Cabib et al., 2023). Currently, there is a dearth of studies that prospectively capture typical labour market exit routes in Sweden by considering different forms of exit at the same time, as well as possible predictors. Furthermore, there are no such studies focusing on high-risk populations, such as individuals placed in OHC during childhood. In the current investigation, we therefore explore labour market exit routes in this particular group, under the assumption that this population has experienced childhood adversity. While the association between childhood adversity and negative outcomes far into adulthood is well documented (Felitti et al., 2019; Jackisch et al., 2019; Kääriälä & Hiilamo, 2017; Sariaslan et al., 2022), labour market exit routes have seldom been considered (Fahy et al., 2017). They can provide important information for understanding the burden faced by this population, since they both reflect previous health and socioeconomic disadvantage, and shape opportunities for healthy ageing. The present study is conducted in a Swedish cohort born in 1953, which allows us to prospectively identify associations between OHC and labour market exit routes. This is done by considering normative (employment to pension) and different types of non-normative exits, such as unemployment or reduced ability to work due to sickness. We view a normative labour market exit as a marker of resilience, i.e., the process of positive adaptation after adversity (Kuh et al., 2003). Conversely, early exit may reflect an accumulation of disadvantages over the life course. Identifying factors that contribute to resilience is crucial, as they buffer long-term harm after childhood adversity and interrupt processes of vulnerability.

### 1.1 Labour market careers from a life-course perspective

Paid work is an important part of life in many ways, and is interrelated with health and well-being. For a large majority of the population, paid work is necessary to ensure a decent standard of living. Ideally, employment is also linked to a sense of belonging and mastery (Moen, 2016). The absence of work comes with negative side effects. Unemployment is likely to be stigmatising since being in paid work throughout midlife is considered normative in most contexts (Brand, 2015; Furaker & Blomsterberg, 2003; Krug et al., 2019). In addition, no work, part-time work, or low-paid work can cause economic insecurity and is associated with poor mental health (Jonsson et al., 2021; Western et al., 2012). Finally, working careers impact finances in retirement, with financial disadvantage potentially forcing the retiree to continue working, and limiting their ability to age in place (Hess et al., 2021; Nygård et al., 2017; Yadav et al., 2023). Due to the bidirectional association between health and work, early labour market exits can signal intersecting health and financial disadvantages, which continue beyond retirement (Halleröd et al., 2013).

Even when in good health and financially secure, retiring from paid work is a significant life transition for many people. Life course theory posits that individuals occupy roles in different domains over their life courses and that transitions between states within these domains can be stressful, depending on timing and context (Elder et al., 2003; Spini et al., 2017). Transitions can be a greater burden when they depart from the socially most common and most accepted timing (Munk, 2014), thereby being non-normative. In general, retirement is often a beneficial transition, for example, for mental health (Lahdenperä et al., 2022; Odone et al., 2021). However, it can constitute a stressful life event under certain circumstances, for example, when financial security is not guaranteed (Barbosa et al., 2016; Lahdenperä et al., 2022). Labour market exits can be considered non-normative if they occur at an atypical age and/or through means other than a standard pension, such as health-related benefits or prolonged unemployment. Accordingly, non-normative exits are often associated with health and financial disadvantages in older age. A better understanding of the life course factors that shape labour market exit patterns, particularly in high-risk groups, is essential for the promotion of healthy ageing in the entire population.

### 1.2 Out-of-home care and other predictors of labour market exit routes

Individuals with experience of childhood adversity are at higher risk of poor educational and labour market outcomes (Hardcastle et al., 2018; Lif et al., 2017). This includes individuals with OHC experience (Brännström, Forsman, et al., 2017; Kääriälä et al., 2019), a group with a particularly high prevalence of childhood adversity (Forsman & Jackisch, 2022; Harris et al., 2025). This is not surprising, since many placements are based on concerns that the family environment does not provide a suitable context for healthy childhood development. This encompasses traditional indicators of childhood adversity, such as parental mental illnesses, including drug abuse disorders, or forms of abuse and neglect (Felitti et al., 2019). In our cohort, all placements before age 13 are placements for family reasons. With increasing age, there are increasingly also placements due to factors related to the child's own behaviour. Children are either placed in foster care or residential care homes. The latter type of placement is meant to provide different treatment options in order to help youth to abstain, for example, from harmful behaviours such as drug misuse and delinquency (Sallnäs, 2009). In summary, the group of placed individuals is highly heterogeneous, both in terms of the underlying reasons for placement, the type of placement, and the length of placement. It is important to acknowledge that OHC placements themselves, irrespective of the decisions behind, by virtue of often incurring a separation from the main caregiver, can be considered an adversity and can entail further adverse experiences related to, for example, placement breakdowns (Briggs-Gowan et al., 2019; Khoo et al., 2012). Given this, OHC placements for family reasons can serve as a valid proxy for childhood adversity in the context of this and similar studies.

Generally speaking, labour market careers, including exit routes, are shaped by health and educational attainment (Avendano & Mackenbach, 2011; Carlsson et al., 2023; Halleröd et al., 2013; Thern et al., 2022). Given that OHC is a known risk factor for both poor health and lower educational attainment (Brännström et al., 2020; Brännström, Forsman, et al., 2017; Forsman, 2020; Kääriälä et al., 2019), it is also likely to predict labour market exit routes. As a consequence, non-normative labour market exits due to, for example, sickness or unemployment, may be more common in individuals with OHC experience. These types of exits can be considered to reflect vulnerability and the underlying accumulation of disadvantage, set in motion by early adversity. There is research linking OHC with disability pension, unemployment, and other forms of socioeconomic and health disadvantage at different ages (Brännström, Vinnerljung, et al., 2017; Harkko et al., 2016).

At the same time, it is important to note that experiencing OHC is not deterministic. Many individuals with experience of childhood adversity, including those who have been placed in OHC, live lives comparable to individuals without such histories (Jäggi et al., 2022; Masten, 2001). This can be viewed as a sign of underlying resilience processes, and it is important to understand how resilience can be fostered in high-risk populations. Higher educational attainment is one possible resilience factor. Since there is an educational gradient in health (Delaruelle et al., 2015), and since higher educational attainment is associated with higher age at retirement (Boissonneault et al., 2020), it generally lowers the risk of early labour market exit. The question is whether higher educational attainment is equally or even more protective in former OHC populations.

Beyond differences by OHC, health, and education, we can also expect gender patterns in labour market exit routes (Madero-Cabib et al., 2023). In the Nordic countries, there is a tendency for women to leave the workforce earlier than men (Madero-Cabib et al., 2023; Soidre, 2005). Work lives are fundamentally gendered, with women more likely to experience fragmented careers due to pregnancy and longer periods of parental leave or other unpaid types of care work (Moen, 2016). These career interruptions increase the risk of economic insecurity by limiting the opportunity to accumulate savings for the future, including for post-retirement, despite women's longer life expectancy (Coppola et al., 2022; Nygård et al., 2017). Therefore, a larger share of women than men experience old-age poverty (Nygård et al., 2017). Since early labour market exit can contribute to this gender gap in income-related old-age poverty, it is important to explore gender patterns in exit trajectories.

To the best of our knowledge, no research to date has taken a person-centred and prospective approach to examining the typical ways in which individuals with OHC experience transition out of the labour market and into retirement. Studies on the long-term consequences of OHC experience or other indicators of childhood adversity in retirement age are often limited by retrospective, self-reported data. The prospective cohort studies that follow individuals with OHC experience often end in midlife. Hitherto, no study has considered multiple early exit routes simultaneously. This study addresses all of these gaps.

### 1.3 The Swedish setting

Given that possible modes of exiting the labour market, as well as possible gender and socioeconomic differences in these exit routes, are determined by structural factors, it is worth dedicating some space to a description of the Swedish context, especially given the welfare reforms occurring over the life course of the cohort members.

The child welfare system, for example, has undergone significant change throughout the years. In the first half of the 20th century, child welfare services did not consider family welfare a primary goal and followed a more interventionist approach than is the case today. During that time, especially the quality of residential placements was increasingly under criticism (Khoo et al., 2012; Sallnäs, 2009). In response, the 1960s and 1970s saw a deinstitutionalization of residential care homes, a prioritization of foster care, and a broader reorientation toward family welfare (Sallnäs, 2009). This occurred alongside an expansion of the welfare state, including child daycare. These developments meant that more individuals – including, for example, single mothers – who might previously have been forced to rely on OHC, could now cope without involvement with child welfare services (Sallnäs, 2009). Taken together, this suggests that the case mix reflected less severe issues during the childhood of the cohort members than can be expected today, because placements have increasingly come to be seen as a last resort. The cohort members grew up during a period of welfare state expansion, but aged in a climate of austerity. Still, despite intensifying cutbacks since the 1990s, Sweden continues to have several comparatively generous measures in place that provide a safety net to its residents. Among them are a largely free education system, affordable childcare, generous parental leave policies, and a largely tax-funded public healthcare system with low out-of-pocket costs (Burström, 2015). Sweden consistently ranks high on gender equality indices (UNDP, 2022), even though there continues to be labour market segregation, with women disproportionately occupying work in the public sector and spending more time in part-time work (Bergqvist, 2016; Hustad et al., 2020; Roeters & Craig, 2014). Overall, Sweden continues to have low absolute poverty rates, but increasing relative poverty rates (Bengtsson et al., 2014; Notten & De Neubourg, 2011).

The Swedish welfare state and its institutions also shape the transition into retirement age, specifically the timing of labour market exits and which types of exit routes are available. The percentage of employed persons aged 55 to 64 varied between 66.2 % and 77.3 % in the years from 2001 to 2021 (SCB, 2024). While there are fluctuations, the trend is going towards higher employment rates in this age group (SCB, 2024). There is no statutory retirement age in Sweden, but the social norm is to retire around the age of 65. The youngest age at which the birth cohort on which this study is based was able to retire was 61. The pension system is earnings-based, but there is a universal minimum amount. In the working-age population, there are two main types of support that persons with health problems can receive to (partially) replace income lost due to a reduced ability to work (SCB, 2019). Firstly, there is disability pension, which is a benefit that can be obtained in cases where there is a likely permanent reduction in the ability to work. Secondly, temporary sickness compensation is available in cases where the individual is likely to return to work later on. When labour force participation is not possible due to a lack of suitable employment options, individuals are entitled to unemployment benefits, conditional upon seeking employment (SCB, 2019).

## 2 Aim and research questions

This study aims to provide a nuanced description of labour market exit routes. We combine sequence analysis with hierarchical clustering to describe typical patterns of exiting the labour market between ages 55 and 68 in a 1953 Swedish birth cohort. We investigate OHC, educational attainment, and gender as predictors of labour market exit routes, while also examining educational attainment and gender as potential effect modifiers of the relationship between OHC and exit routes.

Since individuals with OHC experience often face the dual disadvantage of health-related and socioeconomic vulnerability, we expect greater heterogeneity in labour market exit routes in this population. To capture this, we conduct sequence analysis separately in the smaller subsample comprised only of individuals with OHC experience (placements due to family reasons). A better understanding of exit routes in this group, while considering gender and investigating educational attainment as potential resilience factor, can highlight the complexity of the burden faced by former OHC populations and strengthen arguments for the importance of system- and community-level resilience factors.

The following research questions will be addressed:

RQ1: Considering a range of possible states (health-related benefits, unemployment, pension, and mortality), which non-normative labour market exit routes can be identified in a) the full sample, and b) the sample with OHC experience?
RQ2: In how far do different labour market exit routes correspond to different income profiles, as indicated by average annual disposable income before retirement, and pension after retirement?
RQ3: How are labour market exit routes associated with a) OHC experience, gender, and educational attainment in the full sample, and b) gender and educational attainment in the sample with OHC experience?

## 3 Materials and methods

### 3.1 The SBC multigen

This study is based on the Stockholm Birth Cohort Multigenerational Study (SBC Multigen), which comprises all individuals born in 1953 and living in the greater Stockholm metropolitan area at age 10 (n = 14 608) (Almquist et al., 2019). The follow-up for our study was from age 55 to age 68 (2008–2021) and included all individuals alive at the beginning of follow-up and with sufficient information to determine a labour market state for each year (n = 13 024). For the multinomial logistic regression including education, the sample size was slightly smaller (n = 12 922) due to missing information on that variable. The analyses in the smaller subsample of individuals with OHC experience comprised n = 872 observations, and n = 862 for the multinomial regression models including education.

### 3.2 Context for normative and non-normative labour market exit routes

We consider a transition from employment to retirement, starting at age 61 and beyond, as normative. There is no statutory retirement age in Sweden, but this is the earliest age at which the cohort members could start taking out standard pension, and evidence suggests that early retirement may be associated with health benefits (Hallberg et al., 2015). This type of transition comprises both gradual exits, with a period of receiving income from paid work while also receiving a pension, and instantaneous, directly switching from paid work to retirement pension. Moreover, we consider the receipt of health-related benefits or long-term unemployment before retirement age as non-normative. These exit routes comprise different benefit levels, including situations where income from paid employment is complemented with health-related benefits. Since we include health-related benefits, such as sickness compensation, we do not include health as a separate predictor. It is important to note that the use of the terms “normative” and “non-normative” to categorise labour market exits is not meant prescriptively, designating what a normal exit “should be”. It is rather to, in line with common life course terminology, highlight the penalties incurred by life trajectories departing from social conventions.

Policies surrounding the different types of benefits and pensions have changed over the years, including benefit levels and requirements. Generally speaking, the disability pension (*förtidspension*) system was available until 2003 when the cohort members were 50. The system was then replaced by sickness compensation (*sjukersättning*), where a distinction is made between irreversible reductions in the ability to work and long-term, but time-limited reductions. Despite the name change, the systems work similarly, and individuals become eligible for the respective benefits when there is at least a 25 % reduction in the ability to work (SCB, 2019). Over time, replacement levels have decreased and benefits have become more restrictive across different types of welfare state support, including unemployment benefits (Burström, 2015). The SBC cohort members were furthermore part of a generation with an increasing share of women obtaining higher education and entering the workforce (Thoursie & Wadensjö, 1997).

### 3.3 Variables and measurements

We based the sequence analysis and ultimately, the labour market exit routes, on seven mutually exclusive, binary states, derived from annual information contained in the Longitudinal Integrated Database for Health Insurance and Labour Market Studies (the LISA register). The states were: (i) employment, (ii) pension with income, (iii) pension without income, (iv) unemployment, (v) health-related benefits with income, (vi) health-related benefits without income, (vii) and death. “With income” in states (ii) and (v) refers to years where pension and benefits are complemented by income from an employer, as compared to years where there is no paid employment (states iii and vi). Detailed information is available in the supplementary material (Table S1). In years where more than one state was applicable, we followed a hierarchical coding, with the least normative state taking precedence (vii > vi > v, and so forth). For instance, when a person, within one year, worked, received health-related benefits and then died, death would be the state coded. If the person had returned to work, health-related benefits would still be the state for that year. Information on the year of death was retrieved from the Cause of Death Register.

We furthermore considered the following variables as predictors of different types of labour market exit:

**OHC:** Any placement in residential or foster care due to family reasons (ages 0–19) (any placement/no placement). Generally speaking, placements in foster homes or residential care are mandated in case of threats to the healthy development of the child due to either behavioural problems in the child and/or due to circumstances in the family. In this study, children who have exclusively been placed due to behavioural problems are allocated to the comparison group in the full sample, and not included in the OHC subsample. The decision to not include behavioural placements was made in order to have a robust indicator of severe adversity, assuming that the behavioural placements reflect a higher share of confounding factors independently associated with early labour market exit.

**Gender:** Sex registered at birth (male/female).

**Education:** Highest level of education at age 35 (compulsory/upper secondary or higher).

### 3.4 Statistical analyses

The statistical analysis was conducted in two steps: 1) sequence analysis and hierarchical clustering of labour market exit routes, including the plotting of corresponding income profiles; and 2) using the resulting clusters as outcome in a multinomial regression analysis. Both steps were done separately for the full sample and in the group of individuals with OHC experience. First, labour market exit routes were computed using the states described above. We used the TraMineR package in R (Gabadinho et al., 2011). We plotted the state sequences stratified by OHC experience to provide a visual description of labour market exit routes. Person-centred methods such as sequence analysis have the benefit of retaining heterogeneity across individuals, avoiding to collapse available information into overly simplistic categories right from the start, which variable-centred analyses such as regression models necessarily do. They are data-driven methods that are not superior to regression or time to event models, but provide complementary information. Combined with analyses such as hierarchical clustering, the results can still be sufficiently simplified to enable an exploration of associations between potential predictor variables and common outcome patterns in a population. Elzinga's turbulence was computed to test our assumption of greater heterogeneity in labour market exits in the OHC sample. We then applied hierarchical clustering using the Ward method, both in the full sample (n = 13 024) and stratified by gender (men: n = 6 568, women: n = 6 456), to check for gender patterns in labour market exit routes. We used optimal matching to compute the distance matrix, with an indel cost of 1, and substitution costs calculated based on the transition rates between states. The decision on the number of clusters was based on informativeness when plotting the corresponding chronograms, as well as the PBC, HG and ASW metrics (Studer, 2013). To explore the hypothesis of a greater financial disadvantage in some clusters compared to others, we plotted average annual disposable income, including both income from work and different social benefits after tax, and average annual pension over time. Since some of the clusters, especially the early mortality cluster had too few observations to be representative of that group and their disposable income, we restricted the plot to the time between ages 55 and 64 (62 for the OHC sample). Second, we used the resulting clusters as outcome variables in stepwise multinomial logistic regression. We examined OHC (in the full sample only), educational attainment, and gender as determinants of labour market exit routes. Regression models in the full sample were adjusted for parental socioeconomic status (SES), approximated by the father's occupational status at birth, as a possible confounder of the OHC – labour market exit route association. Data management and regression analyses were done in Stata 18.5, while sequence analyses and hierarchical clustering were conducted in R 4.3.3.

## 4 Results

### 4.1 Labour market exit state sequences and clusters

The full analytical sample consisted of n = 13 024 cohort members (n = 6 568 men, n = 6 456 women). Nearly 7% (n = 451 men, n = 421 women) had been placed in OHC due to family reasons at least once during their childhood (see Table 1).

**Table 1:** Socioeconomic and out-of-home care indicators by sex (count (percentage)).

		Men (n = 6568)	Women (n = 6456)
Education	Upper-secondary education or higher (ref. compulsory education)	5087 (78.1 %)	5416 (84.5 %)
Parental SES∗	Unskilled worker	1274 (20.0 %)	1196 (19.1 %)
	Skilled worker	1795 (28.2 %)	1832 (29.3 %)
	Lower middle class	2407 (37.9 %)	2374 (37.9 %)
	Upper & upper middle class	881 (13.9 %)	856 (13.7 %)
Ever OHC for family reasons		451 (6.9 %)	421 (6.5 %)

∗ Social class based on father's occupation in 1953.

**Fig. 1:**
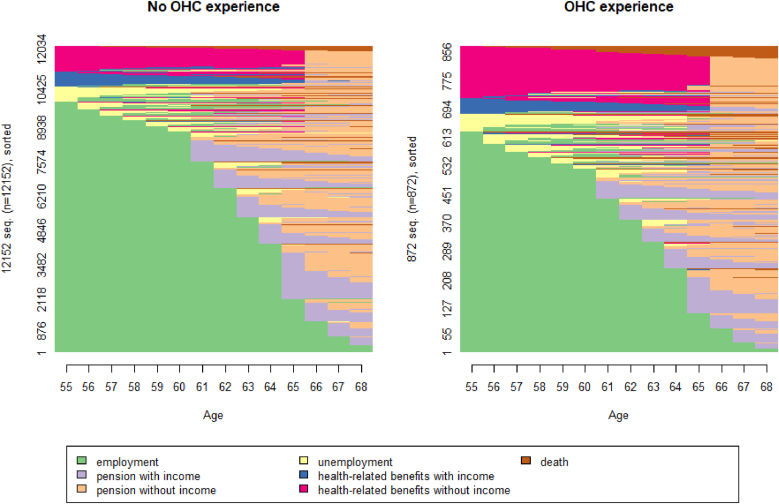
Labour market exit state sequences by OHC experience, sorted by start state (n = 13 024).

The labour market exit state sequences in the full population, stratified by OHC experience, are displayed in Fig. 1. Corresponding figures for the full population, as well as stratified by educational attainment and gender, are available in the supplementary material (Figs. S2–S4).

The most striking difference by OHC is the greater share of health-related benefits (particularly without income) and deaths in those with OHC experience (see also Fig. S5 in the supplementary material for mean time spent in each state), and seemingly greater heterogeneity in the labour market state sequences.

When computing Elzinga's turbulence by OHC experience, the mean turbulence value for individuals with OHC experience (4.65) was higher than in those without OHC experience (4.56), although the difference was not statistically significant (*p* = 0.088). Elzinga's turbulence by education revealed a significantly greater heterogeneity in the group with compulsory education compared to the group with upper secondary or higher education (turbulence values 4.72 and 4.53, *p* < 0.001, Fig. S3). For gender, there was no noteworthy difference in turbulence (*p* = 0.984, Fig. S4).

**Fig. 2:**
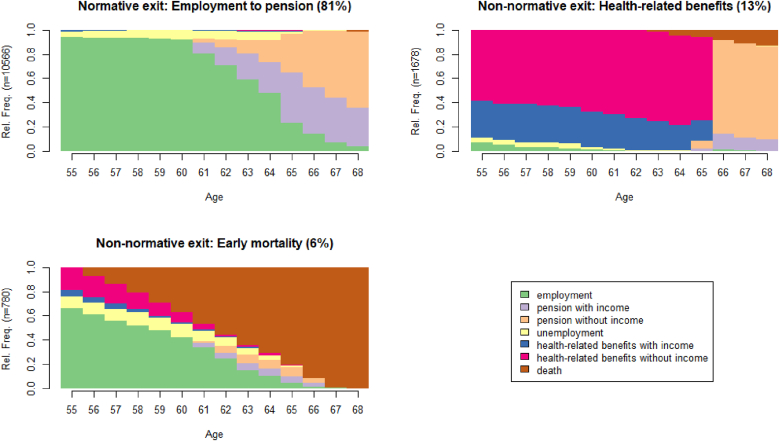
Chronograms for the three-cluster solution in the full population (n = 13 024).

The best fitting clustering for the full sample was achieved using three clusters, the state distribution plots of which are displayed in Fig. 2 (see Table S6 in the supplementary materials for cluster quality metrics and Table S7 for the posterior probabilities for cluster membership by OHC, gender, and educational attainment).

The by far largest cluster represented a *normative exit* (81% in the full sample, 71% among those with OHC experience), characterised by employment followed by a pension. The second largest cluster, *health-related benefits* (13% in the full sample, 21% among those with OHC experience), was characterised by the receipt of different health-related benefits with or without income. Lastly, there was a smaller non-normative cluster characterised by *early mortality* (6% in the full sample, 8% among those with OHC experience). When stratifying by gender, two-cluster solutions fit best in both men and women, resulting in one normative and one non-normative cluster, where the latter combined health-related benefits and early mortality (see Supplementary Fig. S8a and S8b). The normative exit clusters were very similar in men and women, but the prevalence of membership differed (86% of men vs. 81% of women). Regarding the non-normative exit clusters, it is worth noting that women in that cluster spent a larger share of the time obtaining health-related benefits while working, and men had higher mortality during the follow-up.

**Fig. 3:**
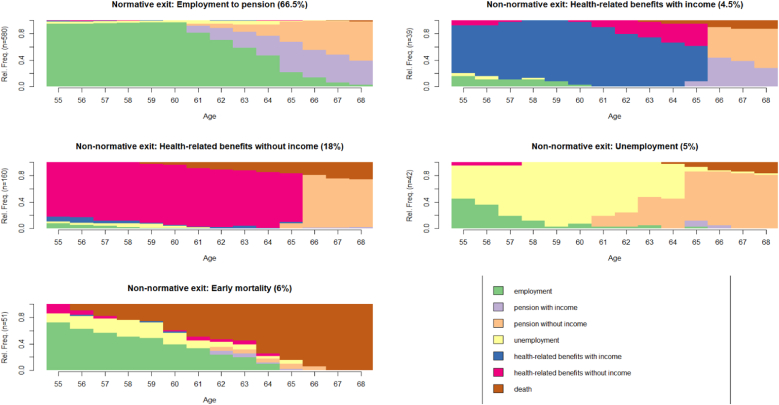
Chronograms for the five-cluster solution in the subsample with OHC experience (n = 872).

The relatively rough clustering in the full sample, combined with the greater share of non-normative exit routes in the population with OHC experience (Fig. 1), suggests that a lot of heterogeneity in labour market exit routes is contained in this smaller subsample. Hierarchical clustering in the sample of OHC-experienced individuals (n = 872) resulted in a five-cluster solution as the best fit (see Table S6 in the supplementary materials for cluster quality metrics). The resulting state distribution plots are presented in Fig. 3.

This clustering provides greater insight into the subtypes of non-normative exits. The majority of the population with OHC experience (66.5%) exited the labour market normatively, moving from *employment to pension*. Furthermore, there were four subtypes of non-normative exit: 4.5% of individuals with OHC experience retired after receiving *health-related benefits with income*. Approximately a fifth of the population with OHC experience (18%) formed a cluster dominated by time with *health-related benefits without income*, followed by a phase of obtaining a retirement pension. Additionally, there was one cluster characterised by a large share of time spent in *unemployment* (5% of individuals with OHC experience), and one cluster, made up of 6% of the subsample, that is characterised by *early mortality*. This means that taken together, 33.5% of the OHC population exited the labour market in ways that signal health and financial disadvantage.

**Fig. 4:**
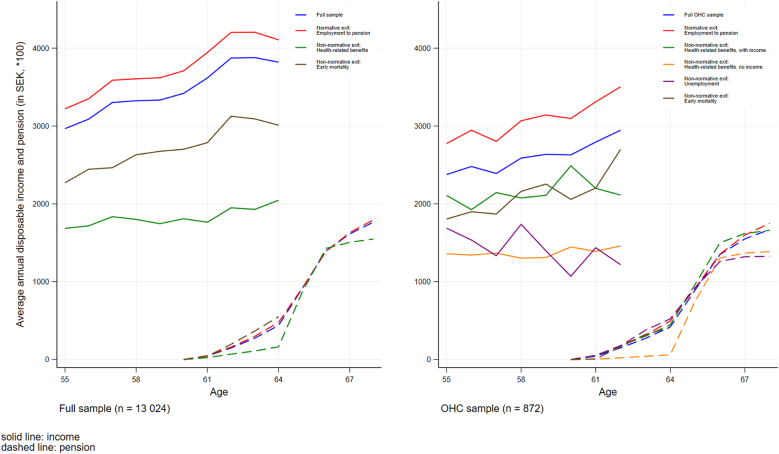
Average annual disposable income and pension levels across labour market exit clusters in the full sample (n = 13 024) and the OHC sample (n = 872).

We plotted average annual disposable income between 2008 and 2017, and pension from 2013 onwards, overall and by cluster, for both the full sample and the OHC sample (Fig. 4). The plot for the full sample shows that the normative labour market exit cluster had the highest disposable income levels, followed by the early mortality cluster. The cluster marked by receipt of health-related benefits had consistently the lowest disposable income levels. Pension levels were relatively even across clusters, except pensions in the health-related benefits cluster, where average pension levels remained lower across most of the follow-up. Overall, average disposable income in the OHC subsample was lower, but again highest in the normative exit cluster. Average disposable income levels were markedly lower in the health-related benefits without income and the unemployment clusters. Pension levels were also noticeably lower in these clusters.

### 4.2 Predictors of labour market exit routes

The results for multinomial logistic regression of labour market exit route clusters on OHC, education and gender in the full sample are reported in Table 2.

**Table 2:** Multinomial logistic regression of labour market exit route clusters on OHC, education, gender, and parental SES (full sample, n = 13 024).

	Model 1		Model 2		Model 3		Model 4	
	OR	95 % CI	OR	95 % CI	OR	95 % CI	OR	95 % CI
Normative exit (base outcome)	1		1		1		1	
Health-related benefits								
OHC (ref. no OHC)	1.93∗∗	1.62, 2.30	1.73∗∗	1.45, 2.07	1.74∗∗	1.45, 2.08	1.71∗∗	1.42, 2.06
Upper-secondary education or higher (ref. compulsory education)			0.46∗∗	0.41, 0.52	0.43∗∗	0.38, 0.49	0.47∗∗	0.41, 0.53
Women (ref. men)					1.73∗∗	1.55, 1.93	1.70∗∗	1.53, 1.90
Parental SES							0.85∗∗	0.81, 0.90
Early mortality								
OHC (ref. no OHC)	1.63∗∗	1.26, 2.11	1.51∗	1.17, 1.95	1.51∗	1.16, 1.95	1.52∗	1.17, 1.98
Upper-secondary education or higher (ref. compulsory education)			0.44∗∗	0.37, 0.51	0.45∗∗	0.39, 0.53	0.46∗∗	0.39, 0.54
Women (ref. men)					0.71∗∗	0.61, 0.82	0.71∗∗	0.61, 0.83

Model 1: Includes OHC; Model 2: Model 1 + education; Model 3: Model 2 + gender; Model 4: Model 3 + parental SES.

Models 2 and 3 comprise n = 12 922 and model 4 n = 12 519 observations due to missing information on the education and parental SES variables.

OHC = out-of-home care.

OR = odds ratio.

Parental SES is a 4-level categorical variable (0 = unskilled worker, 1 = skilled worker, 2 = lower middle-class, 3 = upper & upper middle-class).

∗ p-value <0.05.

∗∗ p-value <0.001.

In the crude model, OHC was associated with an increased likelihood of being in the health-related benefits (OR 1.93, *p* < 0.001) and the early mortality (OR 1.63, *p* < 0.001) cluster, as compared to the normative cluster. This association was attenuated when adding education to the model, but there was very little additional attenuation when adding gender. Finally, we also added parental socioeconomic status to adjust for potential confounding. The full model indicated that OHC increased the odds of membership in both of the non-normative clusters relative to the normative cluster (health-related benefits: OR 1.71, *p* < 0.001, early mortality: OR 1.52, *p* = 0.002). Conversely, having upper secondary or higher education lowered the odds of membership in these clusters (health-related benefits: OR 0.47, *p* < 0.001; early mortality: OR 0.46, *p* < 0.001), as compared to following a normative exit route. Women had a higher likelihood of being in the cluster marked by health-related benefits compared to men (OR 1.70, *p* < 0.001), and a lower likelihood of being in the early mortality cluster (OR 0.71, *p* < 0.001), relative to being in the normative labour market exit cluster. Adding an interaction term between OHC and education, or OHC and gender, did not improve the model fit significantly (*p* = 0.18 and *p* = 0.19, respectively from the post-estimation likelihood ratio test), thus suggesting that there was no effect modification by education.

The results from the multinomial logistic regression in the subsample of individuals with OHC experience, with the corresponding five exit type clusters as the outcome, are presented in Table 3.

**Table 3:** Multinomial logistic regression of labour market exit route clusters on education and gender (OHC subsample, n = 862).

	Model 1		Model 2	
	OR	95 % CI	OR	95 % CI
Normative exit (base outcome)	1		1	
Health-related benefits with income				
Upper-secondary education or higher (ref. compulsory education)	3.02∗	1.06, 8.64	2.89∗	1.01, 8.28
Women (ref. men)			1.77	0.90, 3.48
Health-related benefits without income				
Upper-secondary education or higher (ref. compulsory education)	0.46∗∗	0.32, 0.67	0.46∗∗	0.32, 0.67
Women (ref. men)			0.99	0.69, 1.42
Unemployment				
Upper-secondary education or higher (ref. compulsory education)	0.83	0.41, 1.68	0.84	0.42, 1.69
Women (ref. men)			0.91	0.48, 1.71
Early mortality				
Upper-secondary education or higher (ref. compulsory education)	0.45∗	0.25, 0.82	0.48∗	0.26, 0.86
Women (ref. men)			0.55	0.30, 1.00

Model 1: Includes education; Model 2: Model 1 + gender.

OHC = out-of-home care.

OR = odds ratio.

∗ p-value <0.05.

∗∗ p-value <0.001.

Having at least upper secondary education increased the odds of being in the cluster characterised by health-related benefits with income, relative to the normative exit cluster (OR 2.89, *p* = 0.048). Individuals in this particular cluster were, overall, less financially disadvantaged than individuals in the health-related benefits cluster without income (Fig. 4). Having at least upper secondary education reduced the likelihood of being in the health-related benefits without income and early mortality clusters, relative to the normative exit cluster (OR 0.46, *p* = 0.00 and OR 0.48, *p* = 0.013, respectively). In the smaller subsample with OHC experience, gender was no longer a statistically significant predictor of being in a specific labour market exit cluster. Yet, there was a tendency for women to have a lower risk of being in the early mortality cluster compared to men, relative to the normative exit cluster (OR 0.55, *p* = 0.052).

## 5 Discussion

This study described typical labour market exit routes in a Swedish birth cohort from 1953, both in the overall sample and in the subgroup of individuals with experience of OHC. It furthermore explored gender and education as predictors of labour market exit routes. Three main labour market exit routes were identified in the full sample: one normative exit route of employment followed by pension, and two non-normative exit routes through either receipt of health-related benefits or early mortality. As expected, they represent different degrees of health and financial disadvantage, with average disposable income being distinctly lower in the non-normative clusters. A strong protective factor against non-normative exit routes was upper-secondary education or higher, which was associated with a lower risk of both early mortality and health-related early exits. Women were less likely to be in the early mortality cluster, but more likely to be in the health-related benefits cluster. Our findings show that OHC experience markedly increased the likelihood of being in the two non-normative exit clusters. While over 80 % of the full sample had a normative labour market exit, the corresponding share of those with experiences of OHC was only 66 %. These results highlight that the disadvantages following childhood adversity shape the entire life course. While the majority of individuals with experience of OHC followed normative exit routes, there are very clearly increased risks of non-normative exits.

Non-normative labour market exits indicate an accumulation of disadvantage, keeping the individual locked in a position of vulnerability. This aligns with similar studies from Finland, arguing that these associations are evidence of larger histories of disadvantage, spanning the entirety of a life course (Harkko et al., 2016; Hoven et al., 2018). Non-normative labour market exit routes signal health and socioeconomic disadvantage, potentially driven by health issues in those with early mortality and health-related benefits, and by greater financial disadvantage in those with unemployment and no income to complement social benefits. Such disadvantages are particularly pronounced in the population with OHC experience. We thereby contribute further evidence that OHC, and more generally, childhood adversity, is a risk factor for different, intersecting dimensions of disadvantage.

This study is the first to describe types of non-normative exits in an OHC population, identifying four routes of non-normative exit into retirement age. The increased risk of early mortality affecting individuals with experience of OHC is already well documented (Jackisch et al., 2021; Kääriälä & Hiilamo, 2017). The other three non-normative clusters signal different forms of disadvantage. Early health-related complete exit from the labour market followed by pension was the most prevalent form of non-normative exit among roughly a fifth of the OHC sample. This exit route, together with the unemployment cluster (5%), was marked by the highest financial disadvantage throughout the observation period, including in terms of pension levels. Another 5% of the sample were individuals predominantly receiving health-related benefits with income and then retiring. This group had additional income during their transition into pension and was less financially disadvantaged. Taken together, these findings highlight the importance of approaching the accumulation of disadvantage after childhood adversity holistically, over the life course and under consideration of multiple outcome dimensions.

In adults who are likely to have experienced severe childhood adversity, such as is the case among individuals placed in OHC, normative labour market exit (prevalent in about two-thirds of our sample) can be viewed as a marker of resilience. In this group, education was associated with a decreased risk of some forms of non-normative exit, while gender was no longer a statistically significant predictor of membership in a specific exit route cluster. Education has previously been identified as a mediator of the associations between early adversity and labour market outcomes (Hardcastle et al., 2018; Harkko et al., 2016). Some studies have also investigated education in terms of effect modification in relation to other outcomes, but with mixed results (Afifi et al., 2016; Cosco et al., 2019; Hardcastle et al., 2018). We were interested in the moderating role, but the findings did not support a multiplicative interaction between OHC and education. This means that education is likely equally beneficial among those with and without OHC experiences. Furthermore, the effect of OHC was partially attenuated when adding education to the regression models in the full sample. This indicated that education was an important, but not the only path to disadvantage. For the OHC sample, our study found that attaining upper secondary education lowered the risk of being in the early mortality and health-benefits cluster without income, but was associated with membership in the health-benefits cluster with income. This latter finding was unexpected. It is possible that this route is mainly represented by individuals with white-collar jobs who can reduce their work hours while also receiving a disability pension. However, the results are accompanied by wide confidence intervals, indicating a substantial amount of uncertainty around this estimate due to the small size of this cluster.

Taken together, the findings highlight educational attainment as one factor that can contribute to resilience in former OHC populations. Children placed in OHC are less likely to achieve a level of education that would be protective, because they are disadvantaged from the outset and have a harder time in school. They tend to, for example, have lower educational aspirations and expectations (Kirk et al., 2013), exhibit behavioural and mental health problems that can impact school performance (Lee & Holmes, 2021; Nelson & Gabard-Durnam, 2020), and face additional obstacles such as placement instability (Clemens et al., 2018). To unlock the protective potential of education for more individuals, it seems likely that the school system has to receive additional resources to accommodate different needs better. While this study was not able to provide insights into concrete ways of better catering to the educational needs of OHC populations, there is some existing research on both how schools may in general be able to foster resilience in their students (Twum-Antwi et al., 2020; Ungar et al., 2019), and on how to support OHC populations specifically, even though there seems to be a general lack of promising intervention studies (Berger et al., 2024; Forsman & Vinnerljung, 2012). Furthermore, OHC populations often have to go through an accelerated transition into adulthood, due to the discontinuation of care without a home environment to fall back on while becoming self-sufficient. Measures to help with this transition could include extending placements beyond age 18, improved post-placement support such as continued learning opportunities, and work placements while still in care (Daining & DePanfilis, 2007; Forsman, 2020; Kääriälä et al., 2019; Stewart et al., 2014). These are particularly urgent considerations against the backdrop of growing educational inequalities in labour market exits in Sweden (Almroth et al., 2024).

In terms of differences between men and women, we saw evidence of the frequently observed female-male health-survival paradox in the full sample, with women having more health-related problems and men being overrepresented in the early mortality cluster. In the OHC population, this gender pattern was attenuated. If not due to lack of statistical power, this would indicate that OHC is an overriding risk factor that evens out otherwise observable gender differences. In general, men and women had similar types of labour market exit. This may mean that labour market exits are more homogeneous than mid-life career patterns, where women would typically bear a greater burden of unpaid care work for children and older relatives. Furthermore, labour market exits related to disability pension were historically more prevalent in low-skilled, female-dominated occupations. Today, there is an increasing proportion of men in these jobs (Gonäs et al., 2019), which may, in the meantime, have lowered the gender gap in this type of exit.

It is important to highlight that we based our categorisation of “normative” and “non-normative exit” on obtaining a retirement pension, in contrast to, for example, disability pension. Since there is no statutory retirement age in Sweden, some individuals choose to retire before the normative age of 65. These were considered normative exits, but could still indicate health problems that were not severe enough to warrant a clinical diagnosis and qualify for disability pension. Conversely, there could be many reasons to continue working beyond the normative retirement age. Working beyond retirement can be beneficial and be status or contact-driven, but the possible reasons also include financial necessity (Hess et al., 2021). It would have been preferable to account for workplace characteristics (such as manual vs non-manual labour), which help place retirement decisions into context. We were not able to do so, but the disposable income profile plots suggest that in the non-normative exit clusters, indeed, lower-paid and thereby oftentimes manual work is overrepresented, which may have taken a health toll early on.

### 5.1 Strengths & limitations

This study was able to describe heterogeneity in labour market exit patterns, with a prospective follow-up beyond normative retirement age. We employed multiple different measures of exiting the labour market and had access to a population sample representative of this age group. Furthermore, we had reliable information on the experience of childhood adversity from placement information in municipal child welfare registers, and could thereby investigate labour market exits specifically in a high-risk population. Regarding the use of OHC as an indicator of childhood adversity, it is important to acknowledge that we could not disentangle the effect of placement from the effect of the reasons for placement. Since all of the placement decisions included in the exposure category in this study were made based on problems in the birth family, and since previous research on this cohort has confirmed the suitability of OHC as a proxy for preceding adversity (Jackisch et al., 2019), we are confident that OHC is a suitable indicator of experience of adversity in childhood in the context of this study. Furthermore, the restriction to one set of placement decisions could at least somewhat reduce the already substantial heterogeneity in this group. This is however not to say that only those placed in OHC for family reasons have experienced childhood adversities such as abuse or neglect, or that, conversely, all of them have. To provide more context, Table S9a–S9c in the supplementary material contain information on placement characteristics, highlighting for example the substantial overlap between placement grounds, with nearly half of those placed for behavioural reasons also having experienced placement for family reasons. If we would have included individuals exclusively placed based on behavioural grounds in the OHC population, we would probably have seen an even greater problem burden. The current study design limits the discussion of our findings to a specific category of placement decisions. Another shortcoming is the rough coding of OHC. We could not access detailed information on timing or length, and the OHC category therefore includes a mixture of different types and severities of adversity. Similar to this are the limitations of using sex assigned at birth as a proxy for gender. This study discussed the hypothesised differences between men and women largely in terms of gender, rather than biological sex. We acknowledge that it is not possible to disentangle sex and gender, and that both play a role, but would like to highlight that many labour market outcomes are related to gendered norms around caregiving, as well as labour market segregation. In terms of the labour market states, it would have been preferable to use monthly rather than annual data, for example, about unemployment. We would then have better been able to capture turbulence in labour market exits. We could furthermore not take into account the degree of employment (part-vs full-time), which would have likely resulted in more discernible gender patterns.

Lastly, causal inference in any observational study is challenging, and even more so in studies spanning the largest part of a life course. Our study respected the temporal order of events and there is no obvious way of reverse causation. While there might be unobserved confounding, all associations went in the expected directions and the path between childhood adversity and health or social problems that affect labour market exit is plausible (Bhutta et al., 2023). There are not likely any factors that would have a strong enough relationship with OHC and labour market that they would render our findings invalid. Nevertheless, there are certainly many mediating factors on the pathway between childhood and retirement that would warrant further study. While our findings are not directly translatable to other settings, such as contexts with greater gender inequality, it is likely that the directions of the observed associations – even though maybe not their magnitude – are similar across contexts.

## 6 Conclusions

This study confirmed that populations with a high prevalence of severe childhood adversity, such as former OHC populations, experience intersecting health and socioeconomic disadvantages into retirement. Despite this, a considerable share follows the normative exit route of employment to pension, pointing to underlying resilience processes. Education may play a role in these resilience processes: while attaining upper secondary or higher levels of education did not fully buffer the impact of childhood adversity, it decreased the likelihood of following some of the most disadvantageous exit routes. Future research should identify additional resilience factors in this context. Our results showed that following non-normative labour exit routes may jeopardise well-being after retirement, translating into lower pension levels for some exit route types. Social policies should work to prevent accumulated disadvantage from limiting opportunities for healthy ageing.

## CRediT authorship contribution statement

**Lisa Bornscheuer:** Writing – original draft, Visualization, Methodology, Formal analysis, Conceptualization. **Josephine Jackisch:** Writing – review & editing, Methodology, Conceptualization. **Karl Gauffin:** Writing – review & editing, Supervision, Methodology, Conceptualization. **Ylva B. Almquist:** Writing – review & editing, Supervision, Methodology, Funding acquisition, Data curation, Conceptualization.

## Ethical statement

The Regional Ethical Review Board in Stockholm approved the creation of SBC Multigen (no. 2017/34 − 31/5; 2017/684 − 32). The research project Risk and resilience: Pathways to (ill)health among men and women with experiences of childhood adversity (RISE), of which this study is part, is covered under ethical approval no. 2019–04376.

## Declaration of generative AI and AI-assisted technologies in the writing process

During the preparation of this work the author(s) used ChatGPT in order to proofread the manuscript (spelling, grammar, improve language). After using this tool/service, the author(s) reviewed and edited the content as needed and take(s) full responsibility for the content of the publication.

## Funding

This study was funded by the 10.13039/501100006636Swedish Research Council for Health, Working Life and Welfare (no. 2019-00058).

## Declaration of competing interest

The authors declare that they have no known competing financial interests or personal relationships that could have appeared to influence the work reported in this paper.

## Data Availability

The datasets generated and/or analysed during the current study are not publicly available due to lacking ethical approval for data sharing. Interested researchers are encouraged to directly apply to the registry holders in Sweden.
