# Supplementary material for: Labour market exits in a former out-of-home care population: A birth cohort-based sequence analysis
Source: SSM Popul Health. 2025 Nov 15;32:101885. doi: 10.1016/j.ssmph.2025.101885 (PMC12666444; doi:10.1016/j.ssmph.2025.101885)
Supplement: Multimedia component 1 [file mmc1.docx]

**Supplementary material**

Contents

Table S1: Description of labour market states, including information on underlying variables retrieved from the LISA register (exception: death state; information retrieved from the Cause of Death Register).

Figure S2: Labour market exit sequences, sorted by start state, in the full sample.

Figure S3: Labour market exit sequences by education, sorted by start state.

Figure S4: Labour market exit sequences by gender, sorted by start state.

Figure S5: Mean time (in years) spent in each state, by OHC experience.

Table S6: Clustering quality metrics.

Table S7: Posterior probabilities of cluster membership.

Figure S8a: Labour market exit clusters in men.

Figure S8b: Labour market exit clusters in women.

Tables S9a-c: Absolute frequencies of different types of placement decisions and placement characteristics.

Table S1: Description of labour market states, including information on underlying variables retrieved from the LISA register (exception: death state; information retrieved from the Cause of Death Register).

| **State** | **Description** | **Coded based on** |
| --- | --- | --- |
| Employment | Any amount of income from an employer registered for that year. In some cases, the employment information was complemented using employment status. | LoneInk (gross cash salary) SyssStat (employment status) |
| Pension with income | Any amount of pension registered for that year, plus any amount of income from an employer. | AldPens (sum of age-related pensions)  LoneInk |
| Pension without income | Any amount of pension registered for that year. | AldPens  LoneInk |
| Unemployment | Full- or part-time unemployment benefits received for at least 180 days in a year. Since there were relatively few observations with part-time unemployment, it was considered appropriate to have both full- and part-time unemployment in one category. | ALosDag (days in full-time unemployment)  ADelDag (days in part-time unemployment) |
| Health-related benefits with income | Any amount of sickness compensation received for at least 90 days in that year, and/or any amount of disability pension, while still having an income from work. | ForTid (total income due to early retirement pension/sickness allowance)  SjukErs_Ndag_MiDAS (number of net days with non-time-limited and/or time-limited sickness benefit)  LoneInk |
| Health-related benefits without income | Any amount of sickness compensation received for at least 90 days in that year, and/ or any amount of disability pension, without registered income from work. | ForTid  SjukErs_Ndag_MiDAS  LoneInk |
| Death | If an individual died during follow-up, the state for the year of death was coded accordingly, as were all years following. |  |

If no state could be defined for a given year due to missing information, we used a cruder, but more complete variable on employment status (Sysselsättningsstatus) to complement the information for the employment and unemployment states. Those who were registered as gainfully employed were added to the “employment” state, and those who were not registered as gainfully employed, with or without income statement from the last employer, were added to the “unemployment” state. The cut-offs for sickness compensation and unemployment were chose in line with other studies using the same data sources (Carlsson et al., 2023; Thern et al., 2020, 2022).

**
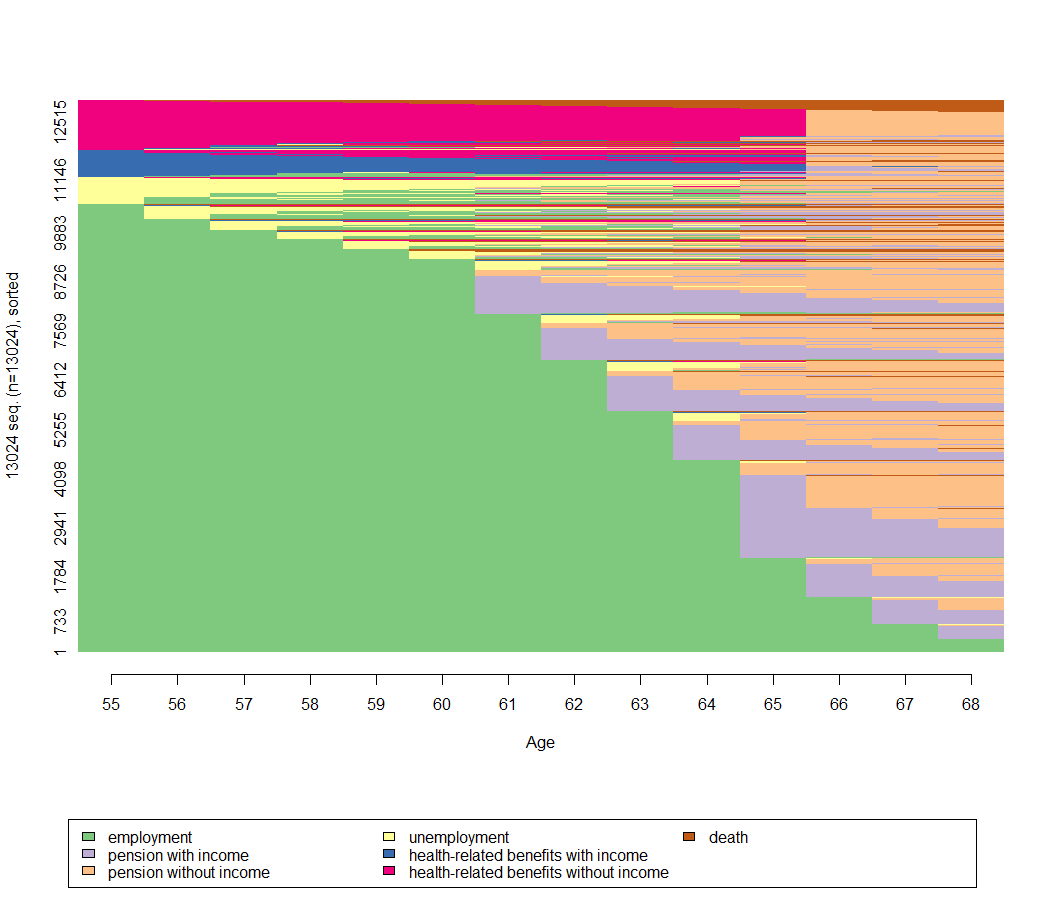
**

Figure S2: Labour market exit sequences, sorted by start state, in the full sample (n=13,024).

**
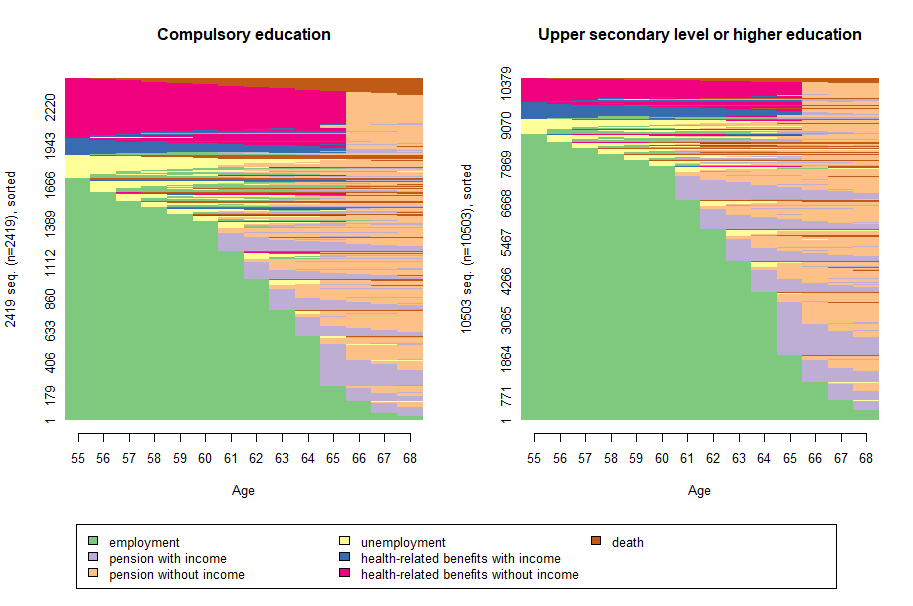
**

Figure S3: Labour market exit sequences by education, sorted by start state. Turbulence values 4.72, compulsory education, and 4.53, upper secondary level or higher education (p < 0.001).


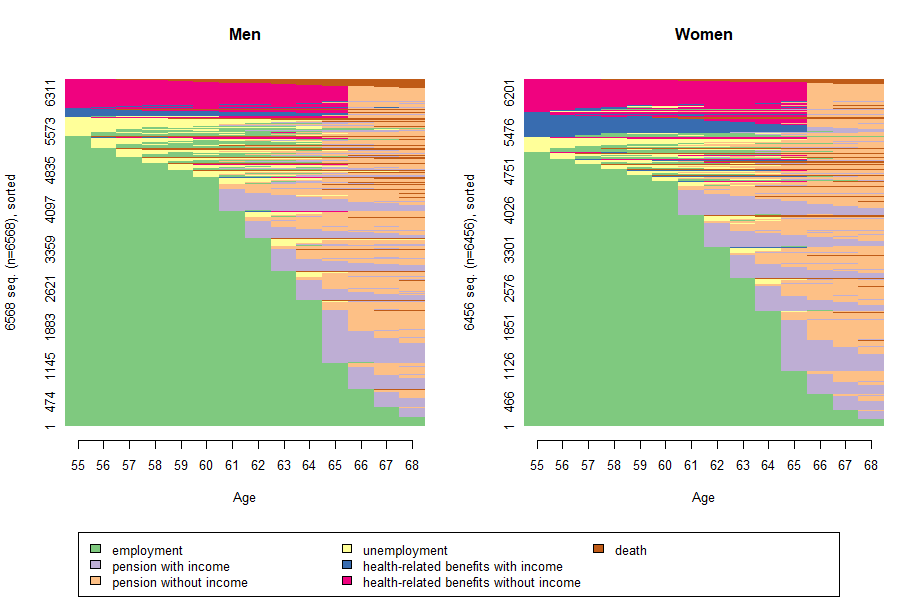


Figure S4: Labour market exit sequences by gender, sorted by start state. Turbulence values 4.57, men, and 4.56, women (p = 0.99).


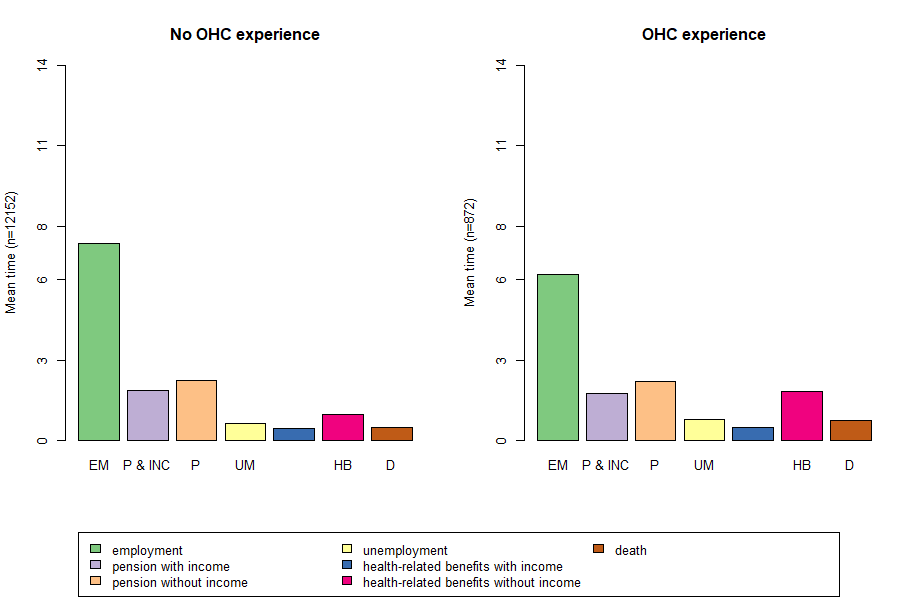


Figure S5: Mean time (in years) spent in each state, by OHC experience.

Table S6: Clustering quality metrics

| **Number of clusters** | **PBC** | | | | **HG** | | | | **ASW** | | | |
| --- | --- | --- | --- | --- | --- | --- | --- | --- | --- | --- | --- | --- |
|  | Full sample | Men | Women | OHC | Full sample | Men | Women | OHC | Full sample | Men | Women | OHC |
| 2 | 0.701 | **0.786** | **0.794** | 0.738 | 0.880 | **0.952** | **0.923** | 0.870 | **0.570** | **0.583** | **0.575** | 0.541 |
| 3 | **0.783** | 0.594 | 0.498 | 0.822 | **0.904** | 0.708 | 0.605 | 0.926 | 0.529 | 0.348 | 0.289 | 0.505 |
| 4 | 0.526 | 0.616 | 0.527 | 0.842 | 0.649 | 0.733 | 0.649 | 0.948 | 0.317 | 0.373 | 0.301 | 0.533 |
| 5 | 0.539 | 0.496 | 0.541 | **0.850** | 0.669 | 0.679 | 0.667 | **0.957** | 0.340 | 0.319 | 0.325 | **0.527** |
| 6 | 0.562 | 0.518 | 0.576 | 0.621 | 0.733 | 0.739 | 0.738 | 0.806 | 0.342 | 0.333 | 0.345 | 0.383 |
| 7 | 0.532 | 0.526 | 0.543 | 0.575 | 0.787 | 0.752 | 0.760 | 0.822 | 0.366 | 0.347 | 0.345 | 0.403 |
| 8 | 0.535 | 0.443 | 0.483 | 0.579 | 0.794 | 0.738 | 0.786 | 0.846 | 0.370 | 0.342 | 0.344 | 0.400 |
| 9 | 0.487 | 0.446 | 0.488 | 0.581 | 0.784 | 0.774 | 0.802 | 0.852 | 0.368 | 0.352 | 0.346 | 0.406 |
| 10 | 0.460 | 0.430 | 0.456 | 0.521 | 0.826 | 0.787 | 0.818 | 0.854 | 0.366 | 0.346 | 0.371 | 0.368 |

Best fit indicated in bold; Full sample: 3 clusters; Men: 2 clusters; Women: 2 clusters; OHC: 5 clusters, PBC: Point Biserial Correlation, HG: Hubert’s Gamma, ASW: Average Silhouette Width; values closer to 1 indicate higher partition quality.

Table S7: Posterior probabilities of cluster membership (mutually adjusted).

| **Probability of cluster membership: Mean (SD); min, max** | | | |
| --- | --- | --- | --- |
|  | Normative exit | Health-related economic disadvantage | Early mortality cluster |
| No OHC experience | 0.82 (0.06); 0.65, 0.88 | 0.12 (0.05); 0.06, 0.28 | 0.06 (0.02); 0.04, 0.12 |
| OHC | 0.71 (0.08); 0.52, 0.82 | 0.21 (0.07); 0.10, 0.38 | 0.09 (0.03); 0.05, 0.15 |
| Compulsory education | 0.70 (0.05); 0.52, 0.78 | 0.20 (0.06); 0.12, 0.38 | 0.10 (0.02); 0.07, 0.15 |
| Upper secondary education or higher | 0.84 (0.03); 0.70, 0.88 | 0.11 (0.03); 0.06, 0.24 | 0.05 (0.01); 0.04, 0.09 |
| Men | 0.83 (0.06); 0.59, 0.88 | 0.10 (0.04); 0.06, 0.26 | 0.07 (0.02); 0.05, 0.15 |
| Women | 0.80 (0.07); 0.52, 0.86 | 0.15 (0.05); 0.11, 0.38 | 0.05 (0.01); 0.04, 0.10 |


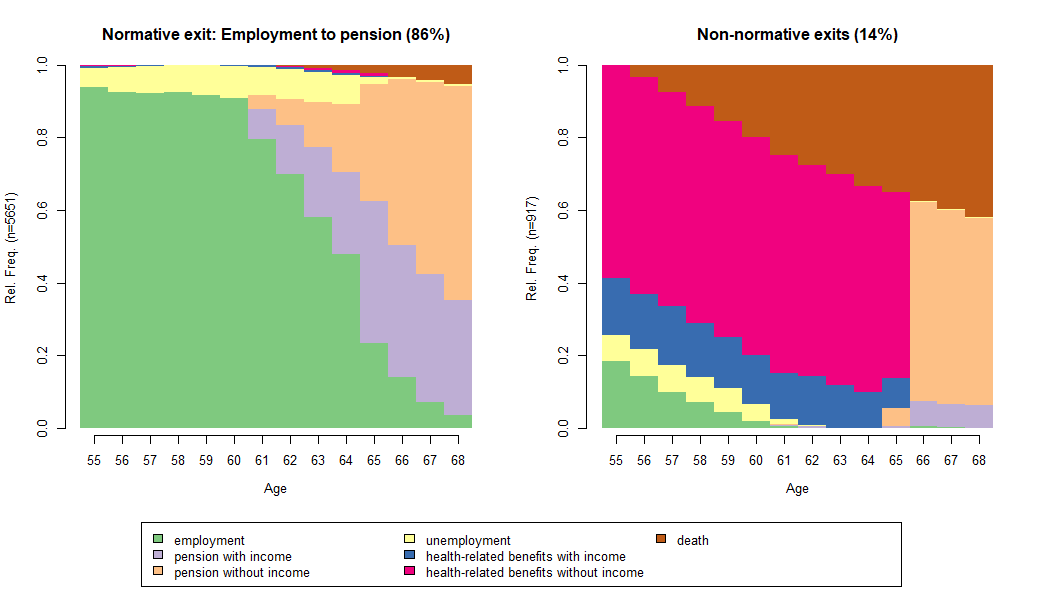


Figure S8a: Labour market exit clusters in men (n = 6 568).


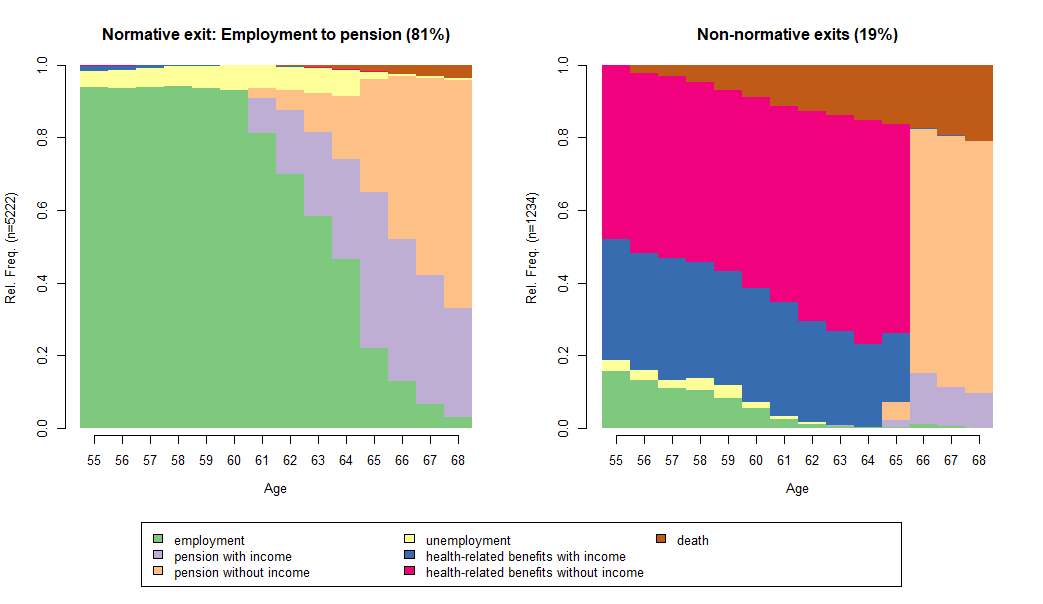


Figure S8b: Labour market exit clusters in women (n = 6 456).

Table S9a: Overlap between placement reasons (family and behavioural reasons)

|  |  | **Ever placed for behavioural reasons** | |
| --- | --- | --- | --- |
|  |  | No | Yes |
| **Ever placed for family reasons** | No | 11,918 | 234 |
|  | Yes | 762 | 110 |

Table S9b: Overlap between placement reasons (family and behavioural reasons) by time period/age group

|  | Period I (age 0-6) | | Period II (age 7-12) | | Period III (age 13-19) | |
| --- | --- | --- | --- | --- | --- | --- |
|  | **OHC (behaviour)**** | | **OHC (behaviour)**** | | **OHC (behaviour)**** | |
| **OHC (family)*** | No | Yes | No | Yes | No | Yes |
| **No** | 12,303 | 0 | 12,775 | 44 | 12,598 | 315 |
| **Yes** | 721 | 0 | 198 | 7 | 98 | 13 |

Table S9c: Frequencies of placement decisions by placement type (foster and residential care) by gender and time period/age group

|  | Period I (age 0-6) | | Period II (age 7-12) | | Period III (age 13-19) | |
| --- | --- | --- | --- | --- | --- | --- |
| **Placement type** | **Boys** | **Girls** | **Boys** | **Girls** | **Boys** | **Girls** |
| **Foster care** | 89 | 106 | 90 | 71 | 146 | 142 |
| **Residential care** | 337 | 276 | 78 | 44 | 144 | 109 |
